# Measurement of Tip Load With a Pacemaker Lead Stylet and Guiding Catheter Using a Silicone Heart Model

**DOI:** 10.7759/cureus.74655

**Published:** 2024-11-28

**Authors:** Daisuke Yamazaki

**Affiliations:** 1 Cardiology, Akita Cerebrospinal and Cardiovascular Center, Akita, JPN

**Keywords:** guiding catheter, his bundle pacing, left bundle blanch area pacing, right ventricular septal pacing, stylet, tip load

## Abstract

Background

Ventricular septal pacing has long been performed using a stylet during pacemaker implantation, but with the availability of guiding catheters, His bundle pacing and left bundle branch area pacing have also been performed. However, it is not known to what extent the tip load of the ventricular lead differs when a guiding catheter is used compared with a stylet alone. In this study, the tip load was measured for different stylet stiffness and guiding catheter geometries at sites where His bundle pacing and left bundle branch area pacing were assumed.

Method

A small weighing instrument was placed in the right ventricular septal portion of the silicone heart model to measure the apical load of the ventricular lead when using two types of guiding catheters and three different types of stylets according to stiffness at the site where His bundle pacing and left bundle branch area pacing were assumed.

Results

The guiding catheter group had by far the highest tip load compared to the stylet group (Site Selective Pacing Catheter (SSPC) multipurpose group 17.9 ± 1.6 g, SSPC extended hook group 3.6 ± 0.8 g). There was no significant difference in tip load between His bundle pacing site and the left bundle branch area pacing site for the normally used stiffness stylet.

Conclusions

Guiding catheters are used for conduction system pacing but must be used with great care as they apply a much greater tip load than conventional lead placement with a stylet alone.

## Introduction

During pacemaker implantation, ventricular leads have traditionally been placed at the apex to facilitate fixation, but apex pacing has been reported to cause dyssynchrony of left ventricular contraction, resulting in reduced contractility and atrial fibrillation [1-2]. Right ventricular septal (RVS) pacing has become common and widespread in the hope of achieving physiological conduction and contraction with the use of screw-in leads and the ability to change the shape of the stylets to select the pacing site. Furthermore, His bundle (HB) pacing and left bundle branch area (LBBA) pacing have been used to achieve more physiological conduction system pacing [3-4]. Delivery catheters are commonly used for HB and LBBA pacing. Ventricular leads include lumenless leads, such as 3830 Select Secure (Medtronic, Columbia, SC), and stylet-driven leads, such as 5076 Capsure Fix Novus MRI (Medtronic, Columbia, SC), 7840 INGEVITY plus (Boston Scientific, Marlborough, MA), 7840 INGEVITY plus (Boston Scientific, Marlborough, MA), Solia S (Biotronik, Berlin, Germany), and Tendril STS (Abbott, Green Oaks, IL). In particular, to perform LBBA pacing, it is necessary to place not only the helix but also a part of the tip of the lead body inside the myocardium. To achieve this regardless of the type of lead used, pressure should be applied to the right ventricular septum while rotating the lead body. It is common to use a guide catheter to advance the lead body into the myocardium because the support provided by the stylet alone is not strong enough. Since there have been no reports on how much the tip load of a ventricular lead differs depending on the stiffness of the stylet and the shape of the guiding catheter when a ventricular lead is implanted, we investigated this issue.

## Materials and methods

**Figure 1:**
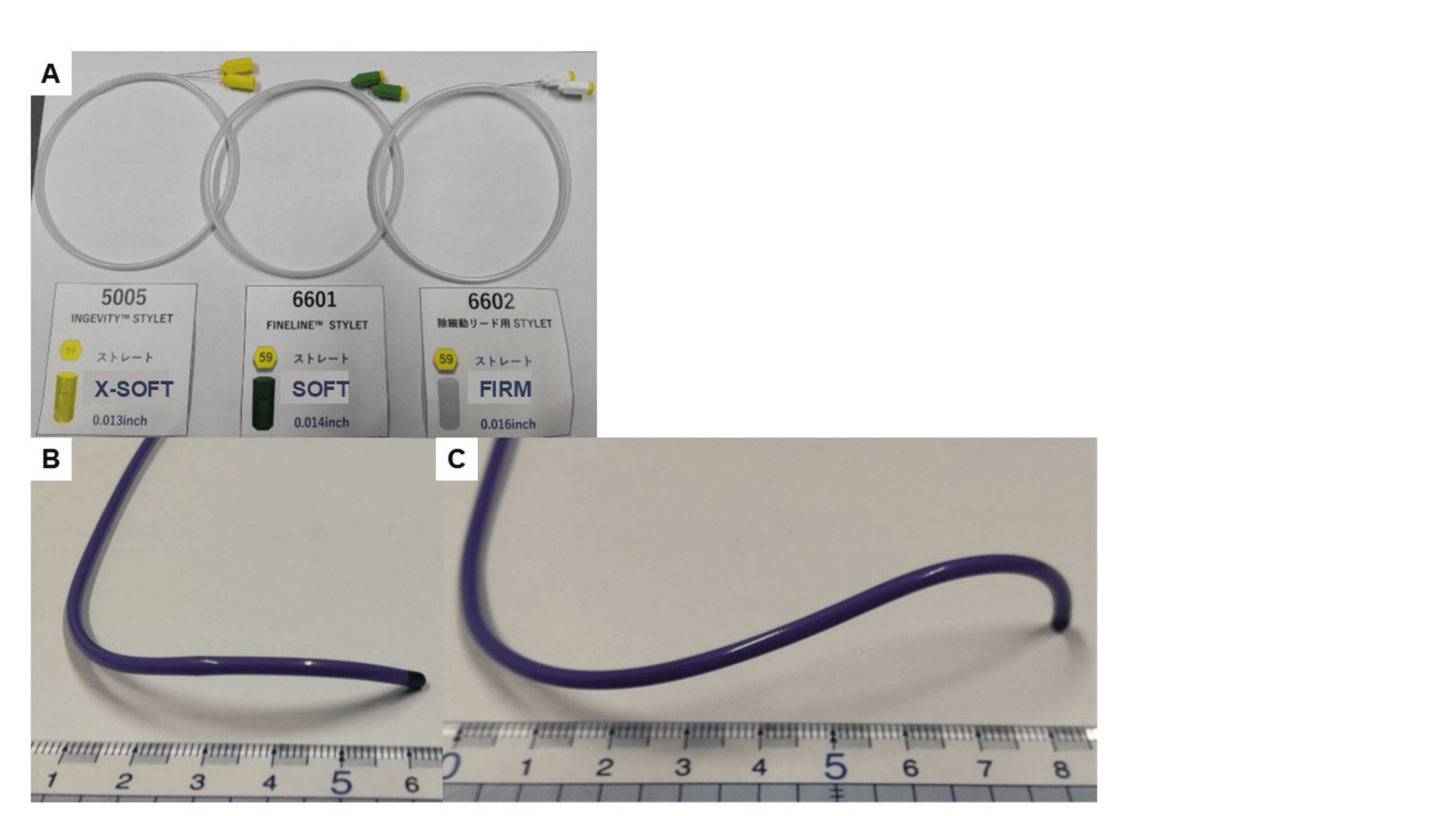
Stylets and guiding catheters used in this study. (A) Three stylet types (X-Soft, Soft, Firm; Boston Scientific, Marlborough, MA). (B) Site Selective Pacing Catheter (SSPC) multipurpose (Boston Scientific, Marlborough, MA) guiding catheter was designed for use in His bundle bunch pacing. (C) SSPC extended hook (Boston Scientific, Marlborough, MA) guiding catheter was designed for use in left bundle brunch area pacing.

The tip load was measured 16 times each with three types of stylets (X-Soft, Soft, Firm; Boston Scientific, Marlborough, MA) (Figure 1A) and two types of delivery catheters (Site Selective Pacing Catheter (SSPC) multipurpose, SSPC extended hook; Boston Scientific, Marlborough, MA) (Figures 1B-1C) at a site representing the usual HB and LBBA.

**Figure 2:**
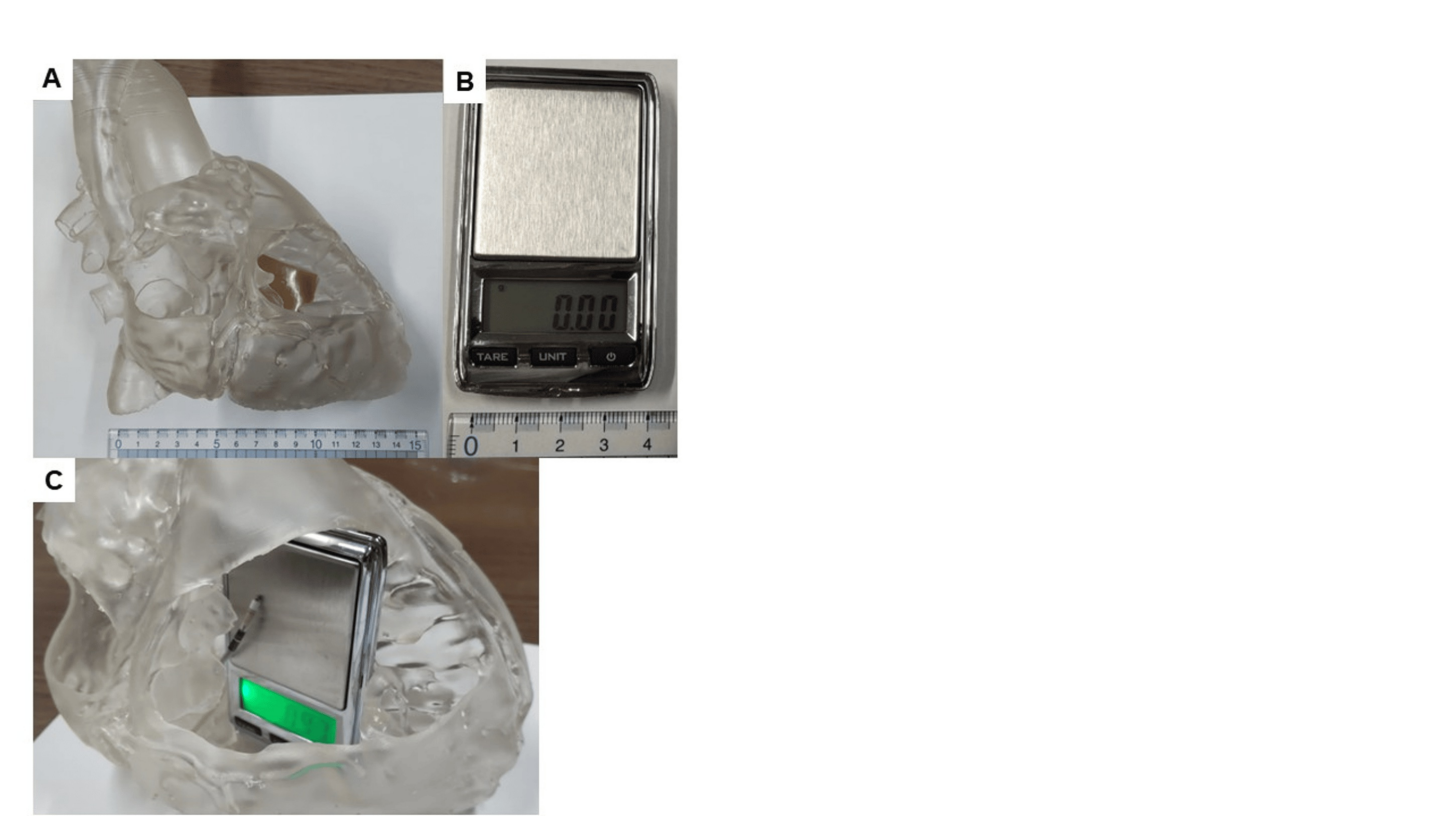
The silicon heart model and small weighing instrument in this study. (A) Silicon heart model. (B) Small weighing instrument. (C) A small weighing instrument was set in the right ventricular septum of the silicone heart model to measure the tip load of the ventricular lead.

In this experiment, we used the silicon heart model in Figure 2A. The tip load was defined as the weight applied to the tip of the lead by placing small weighing instruments (Figure 2B), as shown in Figure 2C, in the RVS of the silicone heart model.

**Figure 3:**
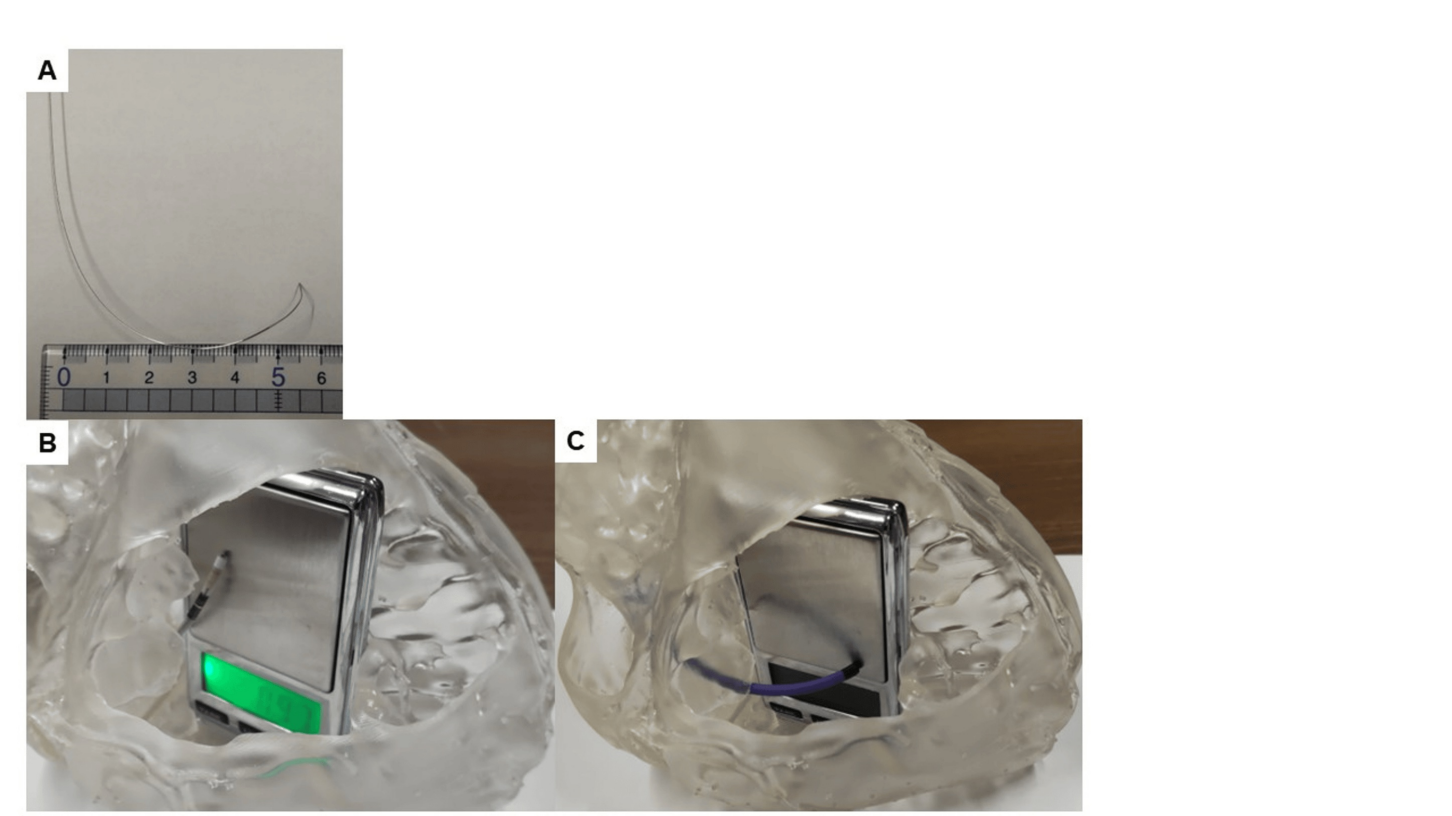
Differences in pacing sites: His bundle area and left bundle brunch area. (A) How to bend the stylet to face the right ventricular septum. After bending into a J-shape, the tip is bent backward about 2 cm so that it faces the ventricular septum. (B) The tip of the ventricular lead is the assumed site for His bundle area. It is in a high position close to the tricuspid annulus. (C) The tip of the guiding catheter is the assumed site for the left bundle brunch area. It is distal and lower than the His bundle area.

The stylet was bent as follows. First, a J-shaped curve was made, and then the tip was bent backward by about 2 cm so that the tip would face the RVS (Figure 3A). When changing the pacing site between the HB and LBBA, the degree of J bending of the stylet was adjusted. The SSPC extended hook was assumed to be used for LBBA pacing, and the SSPC multipurpose was assumed to be used for HB pacing. Figure 3B shows the area assuming HB pacing, and Figure 3C shows the area assuming LBBA pacing. When measuring the tip load, no pushing motion was applied to either the lead stylet or the guiding catheter, but only a counterclockwise torque motion was applied. Torque rotations were performed in the range of 90° to 180°. The lead tip pressed against the right ventricle by the torque motion is shown in Video 1.

**Video 1:** How to apply the tip load. The ventricular lead tip is pressed against the right ventricular septum with a counterclockwise torque.

The tip load measurement group was divided into eight groups: SSPC multipurpose group for HB pacing with guiding catheters, SSPC extended hook group for LBBA pacing, and groups according to stylet stiffness and pacing cite (X-Soft LBBA group, X-Soft HB group, Soft LBBA group, Soft HB group, Firm LBBA group, and Firm HB group). All data were statistically analyzed using the R and R commander-based EZR (Easy R) software (R Foundation for Statistical Computing, Vienna, Austria) [5]. Continuous variables were tested for normality (Kolmogorov-Smirnov test) and presented as mean ± standard deviation if normally distributed or median (interquartile range) if not. The tip loads of the groups were tested by one-way ANOVA and were compared between the groups using the Bonferroni group. A p-value of <0.05 was considered significant.

## Results

**Figure 4:**
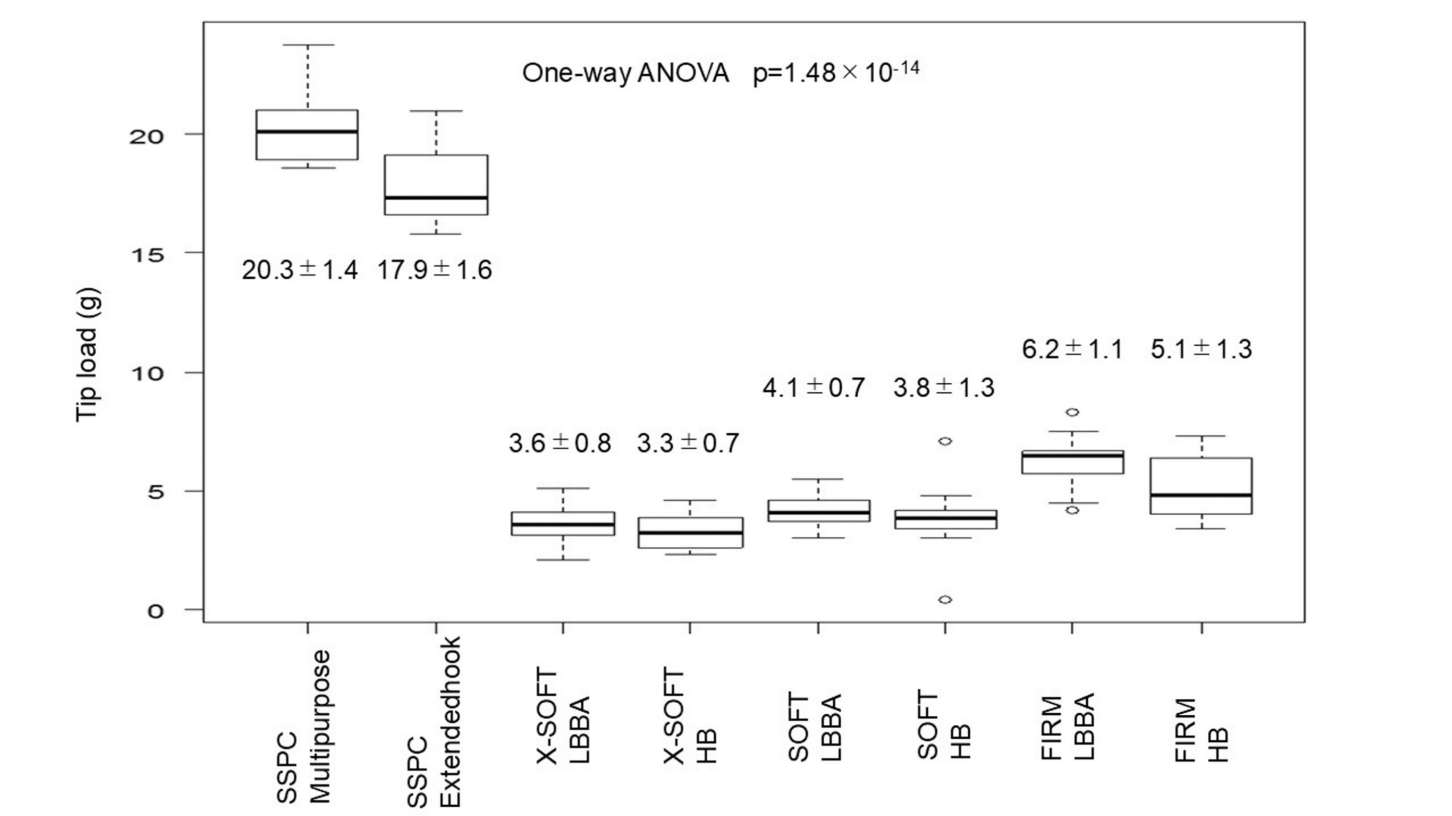
Tip load by pacing site and device. Tip loads (g) grouped by guiding catheter, stylet, and pacing site are shown. It is clear to see that the group of guiding catheters has a higher tip load than the other stylet groups. A p-value of <0.05 was considered a significant difference. HB, His bundle; LBBA, left bundle branch area; RVS, right ventricular septal

Figure 4 shows a graph of the average tip load of two types of guiding catheters and the average tip load of three types of stylets with different hardness in the HB and LBBA regions. The tip loads for each group was 20.3 ± 1.4 g for the SSPC multipurpose group, 17.9 ± 1.6 g for the SSPC extended hook group, 3.6 ± 0.8 g for the X-Soft LBBA group, 3.3 ± 0.7 g for the X-Soft HB group, 4.1 ± 0.7 g for the Soft LBBA group, 3.8 ± 1.3 g for the Soft HB group, 6.2 ± 1.1 g for the Firm LBBA group and 5.1 ± 1.3 g for the Firm HB group. The one-way ANOVA test showed that the tip load was significantly different between groups with p = 1.48 × 10-14, and Figure 4 shows that the tip load with guiding catheters was clearly higher than in the stylet group.

Bonferroni group comparisons are presented in Table 1. The SSPC multipurpose and SSPC extended hook groups had significantly higher tip loads than the other stylet groups. In an intergroup comparison of guiding catheters, the SSPC multipurpose group had a significantly higher tip load than the SSPC extended hook group (20.3 ± 1.4 g vs. 17.9 ± 1.6 g; p = 1.85 × 10-6). The Firm LBBA group had a statistically significantly higher tip load than the other stylet groups; the X-Soft and Soft groups showed no significant difference in tip load regardless of the pacing site. 

**Table 1:** The results of the Bonferroni intergroup comparisons between the respective groups. The guiding catheter group had a significantly higher tip load than the other stylet groups, with the SSPC multipurpose group having the highest tip load. The Firm LBBA group had a significantly higher tip load than the other types of stylet groups. A p-value < 0.05 was considered a significant difference. HB, His bundle; LBBA, left bundle branch area

Group	SSPC multipurpose	SSPC extended hook	X-Soft LBBA	X-Soft HB	Soft LBBA	Soft HB	Firm LBBA
SSPC extended hook	p < 0.001	-	-	-	-	-	-
X-Soft LBBA	p < 0.001	p < 0.001	-	-	-	-	-
X-Soft HB	p < 0.001	p < 0.001	1	-	-	-	-
Soft LBBA	p < 0.001	p < 0.001	1	0.99	-	-	-
Soft HB	p < 0.001	p < 0.001	1	1	1	-	-
Firm LBBA	p < 0.001	p < 0.001	p < 0.001	p < 0.001	p < 0.001	p < 0.001	-
Firm HB	p < 0.001	p < 0.001	0.0099	p < 0.001	0.63	0.08	0.29

## Discussion

RVS pacing became popular because a screw-in lead was developed, and it became possible to select the pacing site by changing the shape of the stylet. In addition, a guiding catheter was developed to achieve more physiological pacing of the conduction system without left ventricular asynchrony [6-7]. The development of guiding catheters has led to HB pacing, but there are also problems, such as high pacing thresholds and difficulty in capturing HB [8]. With improvements in guiding catheter manipulation techniques, LBBA pacing has been attempted by inserting the tip of the lead body into the myocardium [9]. It is difficult to perform HB and LBBA pacing with a stylet alone, and guiding catheters are increasingly needed. However, it was not known how much tip load was applied to the right ventricular wall by the guiding catheters and stylet. Therefore, the tip load was measured in this study. The following are the findings of this experiment.

(i) A much higher tip load can be applied with a guiding catheter than with a stylet alone. (ii) When LBBA pacing is performed with a Firm stylet, higher tip loads can be applied than any other stylet type. (iii) When ventricular leads were placed using a stylet, no significant differences were observed between the HB group and LBBA groups.

In this study, the loading of the lead tip was measured using a silicon heart model and a small measuring device, which has some limitations as it does not faithfully reproduce the actual placement of the lead in vivo. First, in this study, only the torque motion was used to measure the tip load. We also use only torque motion to fix the leads during actual pacemaker implantation. This prevents the lead tip from sliding to the right ventricular outflow tract and slipping into the hinges by a push motion. However, some operators fixate the leads with a push rather than torque motion during lead placement. In the actual right ventricle, there are areas where the tip of the lead does not slide easily because of the trabeculae carneae, allowing the lead to be fixed with a push motion. Therefore, a tip load higher than the current measurements can be expected during lead implantation. The tip load in this study strongly reflects the stiffness of the stylet and guiding catheter, as only torque action was applied.

The second problem is that the experiment using this silicon heart model does not reproduce the state where the helix is fixed within the myocardium. The tip of the screw-in lead is fixed by the helix that penetrates the myocardium. If the lead tip is fixed, the force is transmitted more easily to the lead tip. In the silicon heart model used in this study, it was difficult to reproduce the helix that penetrates the myocardium. Therefore, it is possible to apply higher tip loads in practice. From the above two points, a stronger tip load is expected during lead placement. However, the trend toward much higher tip loads with guiding catheters than with stylets is expected to remain the same.

Among the six groups using the stylet, the Firm LBBA group was able to apply a higher tip load than the other stylet groups; because the Firm stylet is stiffer, there was the impression that its use for lead placement was at risk of perforation. However, the results of this study showed that lead placement with guiding catheters applied a much stronger tip load on the right ventricular wall. Therefore, lead placement using a curved Firm stylet is safer than lead placement using a guiding catheter. However, the diameter of the Firm stylet is increased to make it rigid, so there is almost no space when it is inserted into the lead lumen. For this reason, we had difficulty advancing the Firm stylet into the lead lumen while maintaining its bent shape in the current experiment.

Previous reports have reported perforation of ventricular leads implanted with a conventional stylet [10-11], but as guiding catheters have become more common, there have also been reports of perforation of ventricular leads implanted with guiding catheters. Regarding the type of lead, both lumenless and stylet-driven leads have been reported in ventricular septal perforation [12-13]. There is also a report of perforation of the left ventricular free wall through the ventricular septum after securing the lead to the RVS using a guiding catheter [14]. The tip load of the guiding catheter used in this study was approximately 20 g, which is a level of tip load that is sometimes seen in catheter ablation for the treatment of arrhythmia. However, it is possible that even higher tip loads are actually applied in practice, as discussed above, and case reports on LBBA pacing and perforation using guiding catheters have been published. Therefore, care must be taken when manipulating the guiding catheter during lead placement for LBBA pacing.

Limitations

As mentioned above, the limitation of this study was that it was not possible to reproduce the point where the helix at the tip of the lead was fixed to the myocardium.

## Conclusions

In conclusion, guiding catheters are used for conduction system pacing but must be operated with great care because they apply a much stronger tip load than conventional lead placement with a stylet only.
